# Partial protective efficacy of the current licensed Japanese encephalitis live vaccine against the emerging genotype I Japanese encephalitis virus isolated from sheep

**DOI:** 10.3389/fimmu.2025.1513261

**Published:** 2025-02-13

**Authors:** Hailong Zhang, Yan Zhang, Dan Li, Jiayang Zheng, Junjie Zhang, Zongjie Li, Ke Liu, Beibei Li, Donghua Shao, Yafeng Qiu, Zhiyong Ma, Jianchao Wei, Juxiang Liu

**Affiliations:** ^1^ College of Veterinary Medicine, Hebei Agricultural University, Baoding, China; ^2^ Shanghai Veterinary Research Institute, Chinese Academy of Agricultural Sciences, Shanghai, China

**Keywords:** Japanese encephalitis virus, sheep, SA14-14-2 vaccine, neutralizing antibodies, protective efficacy

## Abstract

**Introduction:**

Vaccination remains the most effective strategy for preventing and controlling Japanese encephalitis (JE). The Japanese encephalitis virus (JEV) seroconversion has been documented in sheep and goats across various countries, with occasional fatal cases occurring among sheep on farms in China. Despite the widespread use of attenuated live vaccines, the efficacy of these vaccines in protecting sheep against JE remains uncertain. This study aimed to assess the protective efficacy of currently available attenuated vaccines against genotype I (GI) JEV strains isolated from sheep using a mouse challenge model.

**Methods:**

In this study, vaccination-challenge experiments were conducted using a mouse challenge model to assess the efficacy of attenuated vaccines. The specific vaccines tested were the SA14-14-2 (GI) and SD12-F120 (GI) attenuated live vaccines. The neutralizing antibodies generated by these vaccines were titrated to evaluate their levels of protection. Mice were immunized with high, medium, or low doses of the vaccines and then challenged with either homologous or heterologous JEV strains. The challenge strains included the SH2201 (GI) and N28 (GIII) strains. Viremia levels and the development of encephalitis lesions were monitored as indicators of protection.

**Results:**

The neutralizing antibody titers against the sheep-derived SH2201 (GI) strain were significantly lower in mice immunized with the SA14-14-2 (GIII) vaccine compared to those receiving the SD12-F120 (GI) vaccine. Immunization with high and medium doses of SA14-14-2 (GIII) vaccine provided complete protection against challenge with the homologous N28 (GIII) strain but only partial protection against the heterologous SH2201 (GI) strain. Mice immunized with medium and low doses of SA14-14-2 (GIII) vaccine showed varying levels of viremia and developed characteristic encephalitis lesions after being challenged with the heterologous SH2201 (GI) strain. Conversely, mice immunized with high and medium doses of the SD12-F120 (GI) vaccine exhibited 100% protection against the challenge with the homologous SH2201 (GI) strain.

**Discussion:**

The results of this study suggest that while the SA14-14-2 (GIII) attenuated live vaccine offers partial protection against sheep-derived GI strains, it is not fully effective against heterologous strains like SH2201 (GI). This highlights a significant gap in the ability of the current vaccines to protect across different JEV genotypes and host species. In contrast, the SD12-F120 (GI) vaccine demonstrated stronger protection against the homologous SH2201 (GI) strain. These findings indicate a pressing need for the development of new vaccination strategies that can provide broader and more effective protection against JE, particularly in diverse host species and against a wide range of JEV genotypes.

## 1 Introduction

Japanese encephalitis (JE) is a zoonotic mosquito-borne viral disease caused by the Japanese encephalitis virus (JEV), first identified in Japan in 1871. The initial Nakayama strain of JEV was isolated from the brain tissue of a deceased patient in 1935 (1, 2). Many species of mammals and birds are susceptible to JEV infection, although most infections remain asymptomatic (3, 4). Clinical symptoms primarily manifest in humans and horses as encephalitis and in pigs as reproductive disorders, with occasional cases reported in other mammals and birds (5, 6). It is estimated that approximately 69,000 cases of JE occur globally each year, with a mortality rate ranging from 20% to 30% (7). Among survivors, approximately 50% are left with permanent sequelae, including severe speech disorders and neurological symptoms. Despite being a vaccine-preventable disease, JE remains endemic in 24 countries and territories across Asia and Oceania, presenting a significant public health challenge of international concern (8).

JEV is a zoonotic flavivirus belonging to the genus Flavivirus in the Flaviviridae family, which comprises more than 70 species including dengue virus, yellow fever virus, West Nile virus, Zika virus and tick-borne encephalitis virus (9, 10). The JEV genome is approximately 11,000 nucleotides long and contains a single open reading frame (ORF) that encodes three structural proteins (C, PrM/M, and E) and seven non-structural proteins (NS1, NS2A, NS2B, NS3, NS4A, NS4B, and NS5) (11–13). Based on the nucleotide sequence of the E gene, JEV is phylogenetically classified into five genotypes (genotypes I - V). From 1935 until the late 1990s, GIII was predominant in several countries, including Japan, South Korea, Thailand, Vietnam, Malaysia, Indonesia, India, Sri Lanka, the Philippines, and Nepal, establishing itself as the dominant genotype in Asia (14). However, recent, molecular epidemiological monitoring has indicated an increase in the number of GI isolates, signaling a transition from GIII to GI (15). In many Asian countries, the re-emerging GI genotype has supplanted GIII as the dominant genotype.

JE vaccination is the most effective measure to prevent JEV infection. There are four different types of JE vaccines available around the world, including mouse brain-derived inactivated, cell culture-derived live-attenuated, cell-culture-derived inactivated, and genetically engineered live-attenuated chimeric vaccines (16). The first licensed JE vaccine was an inactivated mouse brain-derived vaccine based on the Nakayama strain (17), which was highly immunogenic but had drawbacks such as a high production costs and vaccine-induced side effects (16, 18). In China, the Vero cell-derived Beijing-3 vaccine was licensed in 1998 and is currently the main inactivated vaccine used domestically, but it is being replaced by the live attenuated SA14-14-2 vaccine (19, 20). The SA14-14-2 vaccine, a cell culture-derived JE vaccine, was first licensed in China in 1989 and the most widely used JE vaccine in JE-endemic areas (16). Since 2007, China has included the JE vaccine in the National Immunization Program, providing preventive vaccination for children of an appropriate age (1-4 years). There was a significant reduction in morbidity after the inclusion of the JE vaccine in the Expanded Programme on Immunization (4.072/100,000 during 1970-2007 and 0.122/100,000 during 2008-2020) (21). The genetically engineered chimeric live-attenuated vaccine based on YFV 17D as the vaccine vector is now commercially available for use in Australia and Thailand (22).

The E protein, as the principal antigen, plays a crucial role in stimulating the host immune system to produce antibodies (23), aiding in the recognition and elimination of the virus. During viral replication, the E protein is integral to the assembly of viral particles and facilitates their release from the surface of host cells. Mutations at specific amino acid sites within the E protein can influence the virus’s virulence, tropism, and antigenicity (24, 25). In individuals and pigs vaccinated with GIII-derived vaccines, a decline in neutralizing antibody levels against GI strains has been observed (26, 27). Notably, reports from China and India, have documented infections with GI encephalitis strains in patients vaccinated with SA14-14-2 (28, 29), suggesting a reduced protective efficacy of GIII-derived vaccines. Vaccination remains the most effective method for preventing JEV infection (30–32). JEV seroconversion has been documented in sheep and goats across multiple countries (33–35), with sporadic cases reported in sheep farms in China (36). However, the protective efficacy of current Japanese encephalitis vaccines in sheep remains unclear. This study, evaluated the protective efficacy of live-attenuated vaccines against the Japanese encephalitis virus SH2201 strain, isolated from sheep exhibiting neurological symptoms, to assess the vaccines’ effectiveness against emerging sheep-derived JEV strains. The findings will help evaluate existing vaccines’ responses to variant virus strains and provide a scientific foundation for future vaccine development.

## 2 Materials and methods

### 2.1 Ethics statement

All animal experiments were approved by the Institutional Animal Care and Use Committee of the Shanghai Veterinary Research Institute, China (IACUC No: SV-20231229-G03), and were conducted in accordance with the Guidelines on the Humane Treatment of Laboratory Animals (Ministry of Science and Technology of the People’s Republic of China, Policy No. 2006398).

### 2.2 Virus and cells

The JEV genotype I strains SH2201 (GenBank No. PQ488563) and SD12 (GenBank No. MH753127.1), along with the genotype III strain N28 (GenBank No. GQ918133.2) were provided by our laboratory at the Shanghai Veterinary Research Institute. A live-attenuated genotype I strain, SD12-F120 (GenBank No. MN544779), was generated through serial passage of its virulent SD12 strain on BHK-21 cells, combined with multiple plaque purification and virulence selection in mice. The SD12-F120 vaccine strain (GenBank No. MN544780.1) is a vero cell-adapted JEV strain (30). The SA14-14-2 vaccine strain (GenBank No. AF315119.1) was sourced from Wuhan Keqian Biology, Wuhan, China. All JEV strains were propagated and titrated on newborn hamster kidney cells (BHK-21), which were maintained in Dulbecco’s Modified Eagle’s Medium (DMEM; Thermo Fisher Scientific, Carlsbad, CA, USA) supplemented with 10% fetal bovine serum (FBS) at 37°C in a 5% CO_2_ atmosphere. The 50% lethal dose (LD_50_) of each JEV strain was determined using three-week-old female C57BL/6 mice (Shanghai SLAC Laboratory Animal Co. LTD) through intraperitoneal inoculation of serially diluted virus (**Supplementary Table S1**).

### 2.3 Serum samples

A total of 307 sheep serum samples were collected from July to September 2022 from sheep with neurological symptoms (primarily characterized by limb spasms, and ataxia). These samples were provided by the Shanghai Branch of the China Animal Health and Epidemiology Center. Neutralizing antibodies against the JEV SH2201 strain were tested in all 307 serum samples, resulting in the identification of 64 positive serum samples. Subsequently, neutralizing antibodies against the JEV genotype I (SD12) and genotype III (N28) strains were assessed in these 64 positive serum samples.

### 2.4 Multiple amino acid sequence alignment

To perform multiple sequence alignment of the E and NS1 protein amino acid sequence of JEV isolates from sheep, classical JEV strains were retrieved from GenBank using DNASTAR Lasergene 7.1 (MegAlign).

### 2.5 Immunization of animals with SA14-14-2 and SD12-F120 vaccines

Three-week-old female C57BL/6 mice, purchased from Shanghai SLAC Laboratory Animal Co., LTD, were randomly divided into vaccinated and control groups (n = 10 mice/group). The vaccinated groups received intraperitoneally injected of the SA14-14-2 or SD12-F120 vaccine at a dose of 10^5^ PFU per animal. Serum samples were collected 14 days post-vaccination to assess neutralizing antibody titers.

### 2.6 Detection of vaccine protective efficacy in mice

In another experiment, 3-week-old female C57BL/6 mice from Shanghai SLAC Laboratory Animal Co., LTD were also randomly assigned to vaccinated and control groups (n = 15 mice/group). In order to maintain animal welfare and reduce the use of mice, we simultaneously conducted cross protection experiments with the SA14-14-2 and SD12-F120 vaccine strain against the SH2201 strain, and shared data from the JEV strain SH2201, SD12, and N28 strain challenge control groups. Mice were intraperitoneally injected with the SA14-14-2 or SD12-F120 vaccine at doses of 10,000, 1,000 or 100 PFU (plaque-forming unit) per animal. And then were challenged intraperitoneally with a dose of 20 LD_50_ of JEV SH2201 at 14 days post-vaccine boost. The challenged mice were monitored daily for 21 days and the mortality rates were calculated accordingly. Mice blood (n = 5/group) was collected for viraemia detection from day 0 to day 3 post challenge and euthanized on day 7 post challenge and tissue was collected for viral load testing. Histopathologic analysis of brain tissue.

### 2.7 Tissue viral load and histopathological analysis in mice

The viral load of each sample was assessed by RT-qPCR using JEV NS1 gene-specific primers and the viral titer was determined using the TCID_50_ method, as previously described (37). For histopathological analysis, the brain of mice were sectioned and subjected to hematoxylin and eosin staining (Wuhan Servicebio Technology Co., Ltd, Wuhan, China). The severity of histopathological lesions was assessed by determining the integrated optical density in three fields of view using Case Viewer software (Wuhan Servicebio Technology Co., LTD) (38).

### 2.8 Detection of JEV neutralizing antibody titers

Neutralizing antibodies in serum samples collected from both mice and sheep were detected by 50% plaque reduction neutralization test (PRNT_50_), as previously described (39). Briefly, serum samples were inactivated in a water bath at 56˚C for 30 minutes, then serially diluted two-fold, starting from a 1:5 dilution up to 1: 320. The diluted serum samples were mixed with an equal volume of JEV at a concentration of 200 PFU/0.1 ml and incubated at 37˚C for 1 hour. The mixture was then dispensed onto BHK-21 cells grown in 6-well plates and incubated at 37˚C for 2 hours. Following the adsorption period, the cells were overlaid with 1.2% methylcellulose (Thermo Fisher Scientific) in DMEM containing 2% fetal bovine serum (FBS) and incubated at 37˚C for 3 to 5 days until significant viral plaques were observed. The plaques were stained with 0.5% crystal violet, and the neutralizing antibody titers were calculated (40).

### 2.9 Statistical analysis

In this study, the positive cut-off value for neutralizing antibody titers was defined as PRNT_50_ ≥1:10. For PRNT_50_ titers below this positive threshold, the geometric mean titer (GMT) was calculated using a minimum value of 5. All data were processed and analyzed using GraphPad Prism 8.0 software (GraphPad, La Jolla, CA, USA). Student’s t-tests were used for statistical analyses. A *p* value < 0.05 was considered statistically significant.

## 3 Results

### 3.1 Amino acid variations in the E protein between the isolated SH2201 and vaccine strains

We compared the amino acid differences in the E protein between the JEV SH2201 strain, isolated from sheep with neurological signs and genotype I and genotype III vaccine and virulent strains (**Table 1**). The SH2201 strain differs at E138(R→E) from the genotype I live-attenuated vaccine strain SD12-F120. It also exhibits variations at E107 (F→L), E129 (T→M), E138 (K→E), E176 (V→I), E222 (A→S), E244 (G→E), E264 (H→Q), E327 (S→T) and E366 (A→S) compared to the genotype III live-attenuated vaccine strain SD14-14-2 (**Table 1**). Domain III of the E protein is a critical antigenic site antigenic site recognized by the host’s immune system, playing a vital role in generating antibodies against the virus (23). Mutations in Domain III can impact the virus’s virulence, tropism, and antigenicity. Particularly, mutations at E327 (S→T) and E366 (A→S) within Domain III may influence the protective efficacy of the SA14-14-2 vaccine against GI strain infections (39).

**Table 1:** Comparison of amino acid sites of protein E between JEV strains.

JEV strain	Genotype	Amino acids responsible for JEV virulence									
		E107	E123	E129	E138	E176	E222	E244	E264	E327	E366
SH2201	GI	L	S	M	E	I	S	E	Q	T	S
SD12	GI	L	S	M	E	I	S	E	Q	T	S
SD12-F120	GI	L	S	M	R	T	S	E	Q	T	S
NJ2008	GIII	L	R	T	E	I	A	E	Q	S	A
SA14	GIII	L	S	T	E	I	A	E	Q	S	A
SA14-14-2	GIII	F	S	T	K	V	A	G	H	S	A

Additionally, NS1 is a multifunctional protein that plays a role in JEV replication, pathogenesis, and the immune response to infection (41, 42). The SH2201 strain differs from the genotype III live attenuated vaccine strain SA14-14-2 and the genotype I live attenuated vaccine strain SD12-F120 at amino acid position 73 (R→G) and 178 (T→W). The SH2201 strain differs from the genotype I live attenuated vaccine strain SD12-F120 at 219 (G→R) (**Supplementary Figure S1**).

### 3.2 Low titers of neutralizing antibodies to SH2201 stain vaccinated with SA14-14-2 in mice

**Figure 1:**
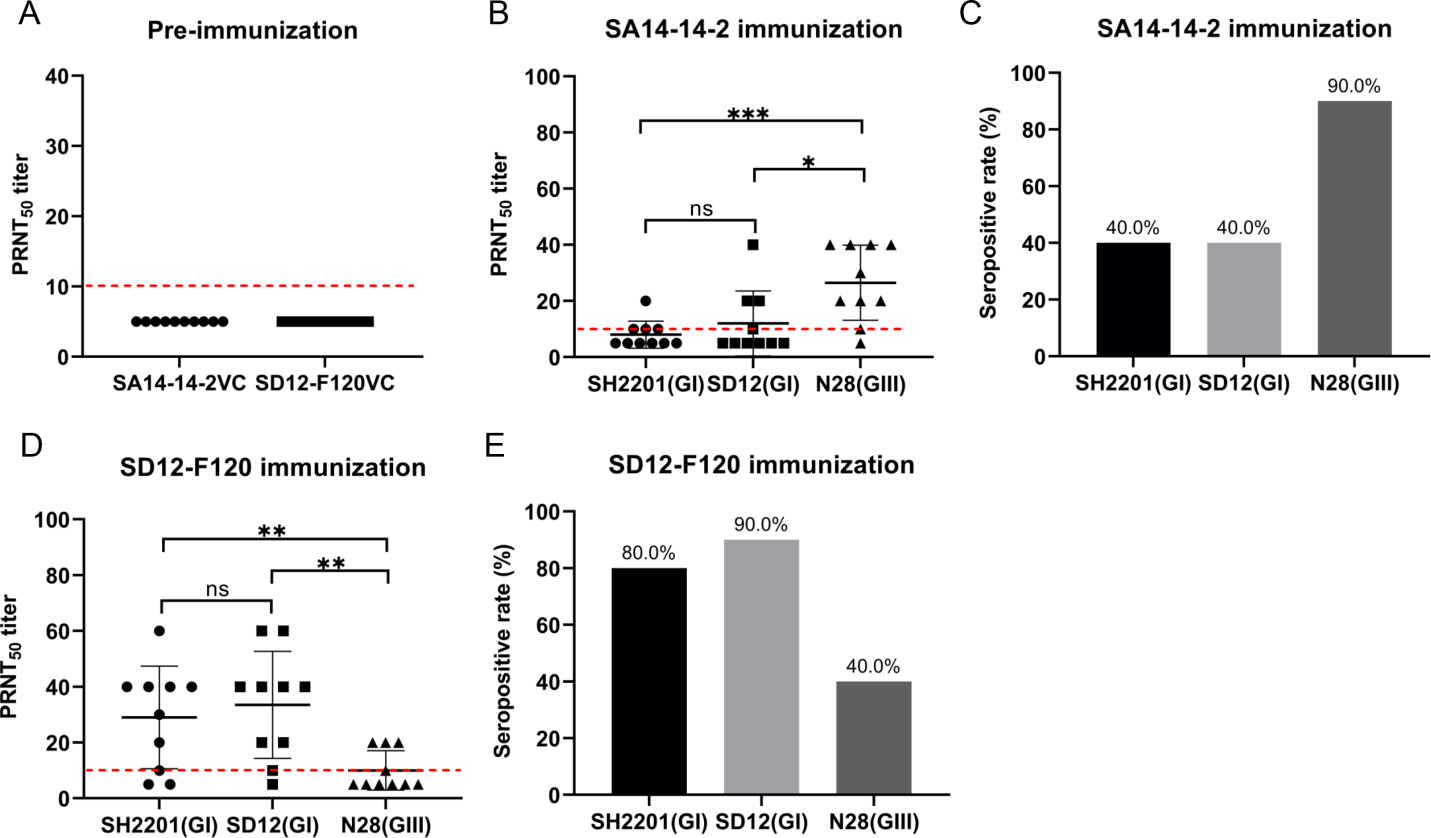
Detection of neutralizing antibody titers in mice vaccinated with the SA14-14-2 and SD12-F120 vaccine. **(A)** Neutralizing antibody titers in mice sera before vaccination with the SA14-14-2 and SD12-F120 vaccine. **(B)** Mice (n = 10) were immunized with SA14-14-2 vaccine and sera for each animal were collected at 14 days post-vaccination for detection of neutralizing antibodies against SH2201(GI), SD12(GI) and N28(GIII) strains. **(C)** Seropositive rate against the indicated JEV strains. **(D)** Mice (n = 10) were immunized with the SD12-F120 vaccine and serum samples were collected at 14 days post-vaccination for detection of neutralizing antibodies against SH2201(GI), SD12 (GI) and N28 (GIII) strains. **(E)** Seropositive rate against the indicated JEV strains. A *p* value was generated by the Student’s t test. ***, *p*<0.001, **, *p*<0.01, *, *p*<0.05. The positive cutoff value (10) for the PRNT_50_ titer is indicated by a red dotted line.

We assessed the neutralizing antibody titers induced by the SA14-14-2 and SD12-F120 vaccines against the sheep-derived SH2201 (GI) strain. Serum samples were collected from mice (n = 10) vaccinated with the SA14-14-2 and SD12-F120 vaccines, and the neutralizing antibody titers (PRNT_50_) specific to the sheep-derived SH2201 (GI), homologous genotype SD12 (GI), and heterologous N28 (GIII) strains were measured. Before vaccination, the neutralizing antibody titers in serum samples were all below 10 (**Figure 1A**). Following vaccination with the SA14-14-2 vaccine, the PRNT_50_ titer against the homologous genotype N28 (GIII) strain was 26.5, higher than the titer (12) for the sheep-derived SH2201 (GI) strain, which had a specificity of 12. The PRNT_50_ titer against the heterologous genotype SD12 (GI) strain was 12, also higher than that for the SH2201 (GI) strain (**Figure 1B**). The seropositivity rate for the homologous N28 strain was 90.0% (9/10), while the seropositivity rates for both the sheep-derived SH2201 (GI) and the heterologous genotype SD12 (GI) strains were 40.0% (4/10) (**Figure 1C**). After vaccination with the SD12-F120 vaccine, the PRNT_50_ titer against the sheep-derived SH2201 (GI) strain was 29, exceeding which was higher than the PRNT_50_ titer of 10 for the heterologous genotype N28 (GIII) strain, but lower than the PRNT_50_ titer of 33.5 for the homologous genotype SD12 (GI) strain (**Figure 1D**). The seropositivity rate for the sheep-derived SH2201 (GI) strain was 80.0% (8/10), while the seropositivity rate for the homologous genotype SD12 (GI) strain was 90.0% (9/10), and the rate for the heterologous genotype N28 (GIII) strain was 40.0% (4/10) (**Figure 1E**). The results above indicate that mice vaccinated with the SA14-14-2 vaccine have a low neutralizing antibody titer against the heterologous genotype SH2201 (GI) strain, with a weaker immune response.

### 3.3 Protective efficacy of SA14-14-2 vaccine against SH2201 strain challenge in mice

**Figure 2:**
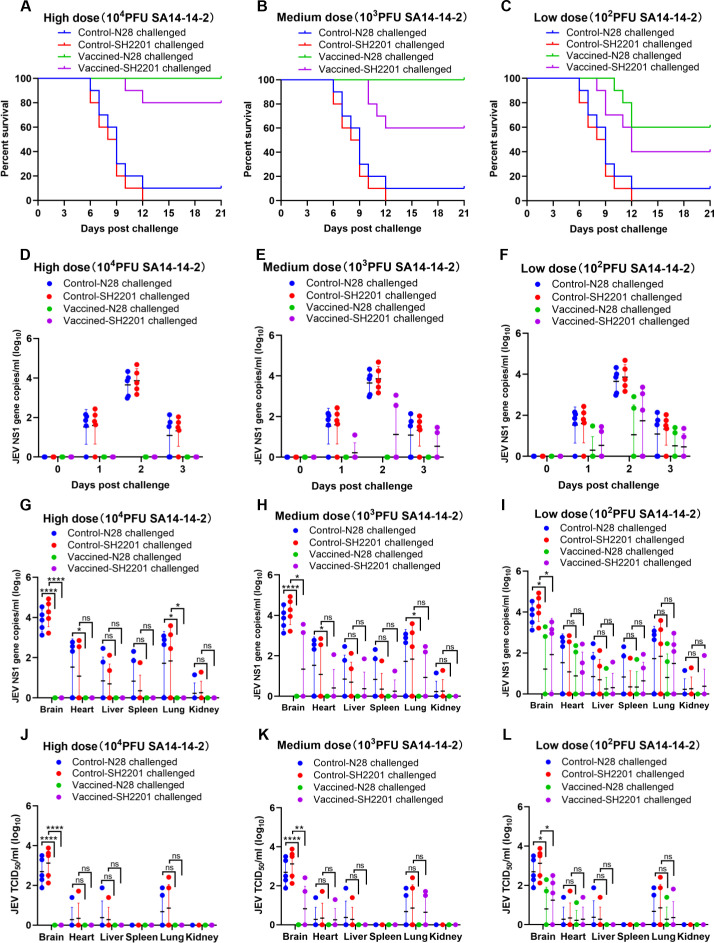
Protective efficacy of the SA14-14-2 vaccine against SH2201 (GI) and N28 (GIII) strains challenge in mice. In order to maintain animal welfare and reduce the use of mice, we conducted cross protection experiments of 3 doses of JEV vaccine SA14-14-2 strain against SH2201 virulent strain simultaneously and shared data from the JEV SH2201 and N28 strains challenge control groups. Each group contained 15 mice, which were divided into the SA14-14-2 high-dose (10,000 PFU), medium-dose (1,000 PFU), and low-dose (100 PFU) vaccine groups, JEV N28 challenge control group, and JEV SH2201 challenge control group. **(A–C)** Mice (n = 10/group) were vaccinated with a mock vaccine (control group) or high-dose (10,000 PFU), medium-dose (1,000 PFU), and low-dose (100 PFU) SA14-14-2 vaccine, and were challenged with the respective JEV strains 14 days post-vaccination. The mice were monitored daily for 21 days to record mortality. Mice (n = 5/group) had blood collected on days 0, 1, 2, and 3 post challenge for detection of RNAemia by RT-qPCR **(D–F)**, and were tested for JEV load by RT-qPCR (G-I) and JEV titers by TCID_50_ **(J–L)** in various tissues on day 7 post challenge. A *p* value was generated by the Student’s t test. ****, *p*<0.0001, **, *p*<0.01, *, *p*<0.05.

Considering that mice vaccinated with the SA14-14-2 vaccine exhibited relatively low titers of specific neutralizing antibodies against the sheep-derived SH2201 (GI), we assessed the protective efficacy of the SA14-14-2 vaccine against challenge with this strain. Mice susceptible to JEV infection were vaccinated with high (10,000 PFU), medium (1,000 PFU), or low (100 PFU) doses of the SA14-14-2 vaccine, and then were challenged intraperitoneally with a dose of 20 LD_50_ of the sheep-derived SH2201 (GI) strain or the homologous genotype N28 (GIII) strain. The results demonstrated that mice vaccinated with high and medium doses of the SA14-14-2 vaccine were completely protected from the homologous genotype N28 (GIII) strain. However, those receiving a low dose showed only partial protection, achieving a survival rate of 60.0% (**Figure 2C**). Furthermore, none of the dosage groups provided complete protection against the sheep-derived SH2201 (GI) strain, with protection rates of 80%, 60%, and 40% observed for the high, medium, and low dose groups, respectively (**Figures 2A-C**).

For additional validation of the immunoprotective effect of the SA14-14-2 live-attenuated vaccine, blood was collected from mice on day 0 to day 3 post challenge to measure the viremia. The results showed that the viremia in the N28 and SH2201 strain control group mice peaked on day 2 post-challenge, and viremia up to 2.1×10^4^ RNA copies/ml to 4.8×10^4^ RNA copies/ml (**Figures 2D–F**). No viremia was detected in the high and medium-dose SA14-14-2 vaccine groups following challenge with the homologous N28 strain (**Figures 2D, E**). In the low-dose group, viremia was detected in 2 out of the mice challenged with the homologous N28 strain (**Figures 2F**). For the challenge with the heterologous SH2201 strain, viremia was detected in 2 mice in the medium-dose vaccine group and in 3 mice in the low-dose group (**Figures 2E, F**).

Additionally, we also measured the viral load and titer in various tissues of the control and vaccine-immunized mice. The results showed that the highest viral loads were observed in the brains of N28 and SH2201 control mice, with titers ranging from 7.6×10^1^ to 7.6×10^3^ TCID_50_/ml. Other tissues (heart, liver, spleen, lung and kidney) were also detected at different levels of viral load (**Figures 2G–L**). JEV was not detected in the tissues of mice challenged with homozygous N28 in the high-dose and medium-dose SA14-14-2 vaccine immunization groups (**Figures 2G, H**). In the low-dose group, JEV was detected in the brain, heart, liver, spleen, and lung tissues of two mice challenged with the homologous N28 strain, with the highest viral titer in the brain, ranging from 5.1×10^1^ to 1.9×10^2^ TCID_50_/ml (**Figures 2I, L**). In the medium and low-dose vaccine groups, 2-3 mice had JEV detected in the brain, heart, liver, spleen, and lung tissues after challenge with the heterologous SH2201 strain (**Figures 2H, I**), with the highest viral titer in the brain tissue of 3.2×10^2^ TCID_50_/ml (**Figures 2K, L**). A significant difference in the viral titer in the brain tissue was observed between the high and medium-dose vaccine groups and the control group. These data indicate that the SA14-14-2 vaccine offers partial protection against the sheep-derived SH2201 (GI) strain.

### 3.4 Protective efficacy of SD12-F120 vaccine against SH2201 strain challenge in mice

**Figure 3:**
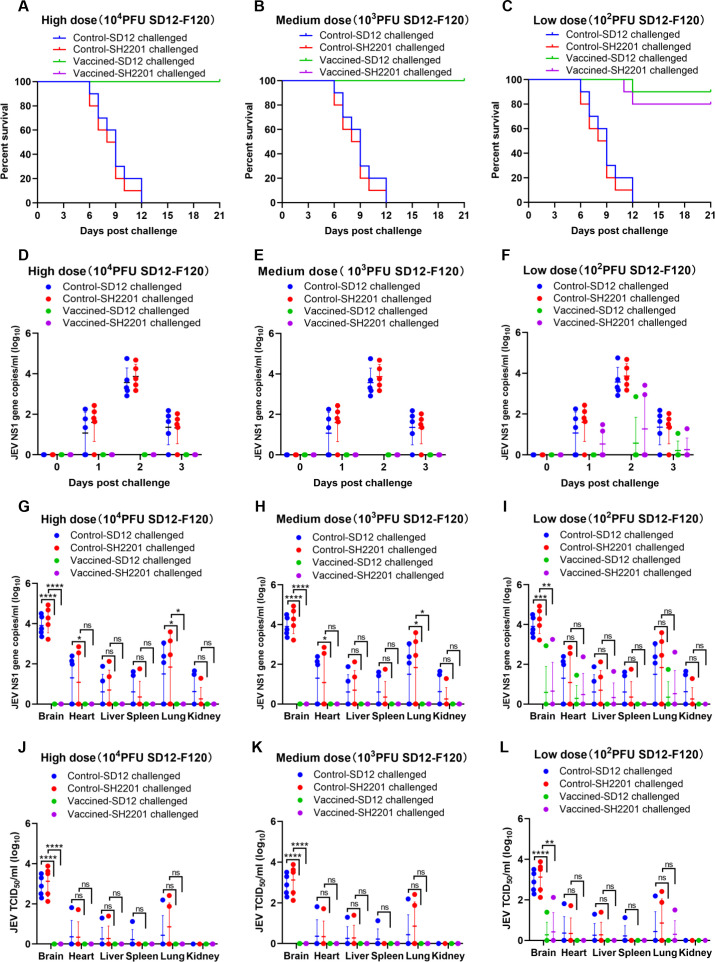
Protective efficacy of the SD12-F120 vaccine against SH2201 (GI) and SD12 (GI) strains challenge in mice. In order to maintain animal welfare and reduce the use of mice, we conducted cross protection experiments of 3 doses of JEV vaccine SD12-F120 strain against SH2201 virulent strain simultaneously and shared the data of JEV SD12 and SH2201 strains in the challenge control group. Each group contained 15 mice, which were divided into the SD12-F120 high-dose (10,000 PFU), medium-dose (1,000 PFU), and low-dose (100 PFU) vaccine groups, JEV SD12 challenge control group, and JEV SH2201 challenge control group. **(A-C)** Mice (n = 10/group) were vaccinated with a mock vaccine (control group) or high-dose (10,000 PFU), medium-dose (1,000 PFU), and low-dose (100 PFU) SD12-F120 vaccine, and were challenged with the respective JEV strains 14 days post-vaccination. The mice were monitored daily for 21 days to record mortality. Mice (n = 5/group) had blood collected on days 0, 1, 2, and 3 post challenge for detection of RNAemia by RT-qPCR **(D-F)**, and were tested for JEV load by RT-qPCR **(G-I)** and JEV titers by TCID_50_ **(J-L)** in various tissues on day 7 post challenge. A *p* value was generated by the Student’s t test. ****p<0.0001, ***p<0.001, **p<0.01, *p<0.05.

The development of genotype I (GI) vaccine has been proposed to control GI encephalitis virus infections, as the genotype III (GIII) encephalitis vaccine has been shown to lack complete cross-protection against GI strains (43). Given that the JEV strain SH2201, isolated from neurologically symptomatic sheep, belongs to genotype I, we evaluated the protective efficacy of the SD12-F120 (GI) vaccine against challenge with the SH2201 (GI) virus. Mice were vaccinated with high (10,000 PFU), medium (1,000 PFU), or low (100 PFU) doses of the SD12-F120 (GI) vaccine and then challenged with both homologous SD12 (GI) and sheep-derived SH2201 (GI) viruses. The high and medium doses of the SD12-F120 vaccine provided complete protection against both the homologous SD12 (GI) and the sheep-derived SH2201 (GI) strains (**Figures 3A, B**). However, the low dose did not fully protect the mice, yielding protection rates of 90.0% against SD12 (GI) and 80.0% against SH2201 (GI) (**Figure 3C**).

For additional validation of the immunoprotective effect of the SD12-F120 live-attenuated vaccine, blood was collected from mice on day 0 to day 3 post challenge to measure the viremia. The results showed that the viremia in the SH2201 and SD12 strains control group mice peaked on day 2 post-challenge, and viremia up to 4.8×10^4^ RNA copies/mL to 5.6×10^4^ RNA copies/ml (**Figures 3D–F**). No viremia was detected in the high and medium-dose SD12-F120 vaccine groups following challenge with the homologous SD12 and SH2201 strain (**Figures 3D, E**). In the low-dose group, viremia was detected in one out of the mice challenged with the homologous SD12 strain and two out of the mice challenged with the homologous SH2201 strain (**Figures 3F**).

Additionally, we also measured the viral load and titer in various tissues of the control and vaccine-immunized mice. The results showed that the highest viral loads were observed in the brains of SD12 and SH2201 control mice, with titers ranging from 1.3×10^2^ to 7.6×10^3^ TCID_50_/ml. Other tissues (heart, liver, spleen, lung and kidney) were also detected at different levels of viral load (**Figures 3G–L**). JEV was not detected in the tissues of mice challenged with homozygous SD12 and SH2201 in the high-dose and medium-dose SD12-F120 vaccine immunization groups (**Figures 3G, H**). In the low-dose group, JEV was detected in the brain, heart, and lung tissues of one mouse challenged with the homologous SD12 strain, with the highest viral titer was 2.5×10^1^ TCID_50_/ml in the brain. And in the brain, heart, liver, and lung tissues of one mouse challenged with the homologous SH2201 strain, with the highest viral titer was 1.3×10^2^ TCID_50_/ml in the brain (**Figures 3I, L**). A significant difference in the viral titer in the brain tissue was observed between vaccine immunization groups and the control group. These results demonstrate that the SD12-F120 vaccine offers substantial protective efficacy against the sheep-derived SH2201 (GI) strain, achieving over 80.0% protection even at low doses.

### 3.5 Histopathological lesions in brains of vaccinated mice

**Figure 4:**
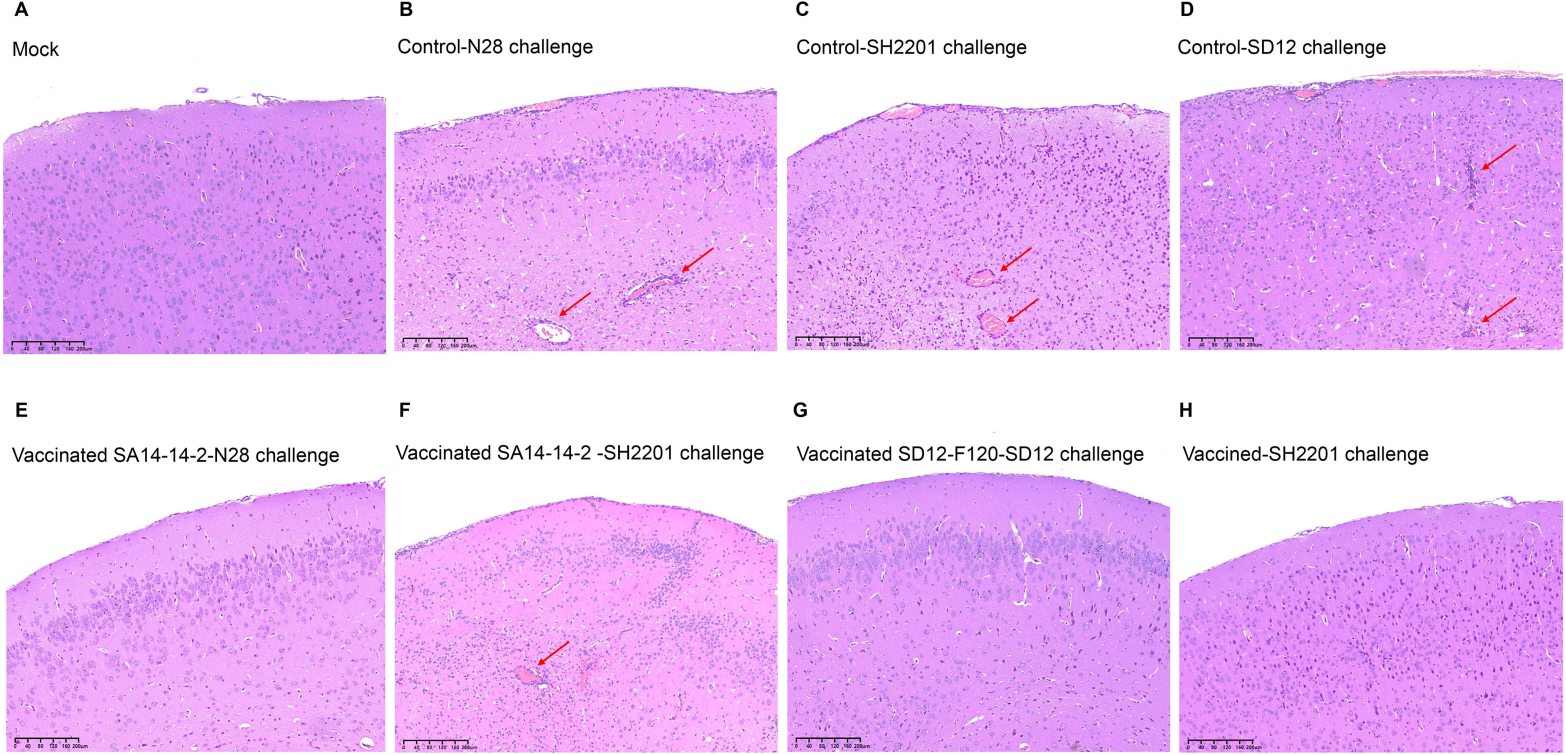
Histopathological lesions in brains of vaccinated mice. Mice (n = 5) were immunized with SA14-14-2 or SD12-F120 at doses of 1,000 PFU per mouse and challenged with the N28, SH2201 or SD12 strains at 14 days post vaccination. Brain samples were collected at 7 days post challenge for analysis of histopathological lesions. The sectioned brain simples were stained with hematoxylin and eosin. The multifocal lymphohistiocytic perivascular cuffs are indicated by arrows. **(A-D)** Histopathological lesions in brain samples collected from mock mice challenged with N28 (GIII), SH2201(GI), and SD12 (GI) strains. **(E, F)** Histopathological lesions in brain samples collected from SA14-14-2 (GIII) vaccinated mice challenged with N28 (GIII) and SH2201 strains. **(G, H)** Histopathological lesions in brain samples collected from SD12-F120 (GI) vaccinated mice challenged with SD12(GI) and SH2201(GI) strains.

To further explore the cross-protection, brain samples were collected from the vaccinated and control groups mice at 7 days post challenge for analysis of histopathological lesions. The histopathological lesions of encephalitis were not observed in blank control mice, whereas the control mice challenged with the virulent N28 (GIII), SH2201 (GI), or SD12 (GI) strain developed encephalitis histopathologically characterized by the multifocal lymphohistiocytic (**Figures 4A–D**). The histopathological lesions of encephalitis were not observed in the brain tissue of mice from the SA14-14-2 vaccine immunization group challenged with the homologous N28 (GIII) strain. However, histopathological lesions of encephalitis were observed of mice challenged with the heterologous SH2201 (GI) strain (**Figures 4E, F**). The histopathological lesions of encephalitis were not observed in the brain tissue of mice from the SD12-F120 vaccine immunization group challenged with either the homologous SD12 (GI) or the SH2201 (GI) strain (**Figures 4G, H**).

### 3.6 Detection of neutralizing antibodies against SH2201 JEV strain in sheep sera with Japanese encephalitis

Neutralizing antibody titers (expressed as GMT) were assessed in 64 serum samples from convalescent sheep with Japanese encephalitis. The results indicated that when the neutralizing antibody titer against the SH2201 strain ranged from 1:10 to 1:20, there was protective efficacy against the SD12 (GI) strain (GMT ≥ 10). When the neutralizing antibody titer against SH2201 reached ≥ 1:40 (GMT ≥ 10), protective efficacy was observed against both the SD12 (GI) and N28 (GIII) strains, with serum positivity rates of 100% for both strains (**Table 2**). These findings confirm the presence of a certain degree of JEV infection and prevalence among sheep in clinical settings.

**Table 2:** Geometric mean titers (GMT) of neutralizing antibody titers to SH2201 strain in sheep sera with Japanese encephalitis.

PRNT_50_ against SH2201	Sample size	PRNT_50_ GMT against JEV (95% CI)			Seropositivity rate against JEV (%)*		
		SH2201(GI)	SD12(GI)	N28(GIII)	SH2201(GI)	SD12(GI)	N28(GIII)
10-20	19	15.5	13.2	7.9	100.0	100.0	57.9
40-80	34	56.2	42.7	36.3	100.0	100.0	100.0
≥160	11	158.4	89.1	47.9	100.0	100.0	100.0
Total	64	45.7	32.4	24.0	100.0	100.0	86.0

*Defined as plaque reduction neutralization test (PRNT_50_) ≥ 1:10 using BHK-21 cells.

## 4 Discussion

The host range of Japanese encephalitis is extensive, capable of infecting various mammals. Infection in pregnant sows can result in abortion and stillbirths (44). While horses typically experience subclinical infections, some may exhibit fever, anorexia, and dyskinesia (45). Additionally, JEV seroconversion has been reported in sheep and goats in multiple countries (33–35), with sporadic infections noted in sheep farms in China (36), leading to certain economic losses for those operations.

There are currently no specific antiviral drugs available for treating Japanese encephalitis virus (JEV) infections. In the absence of effective antiviral interventions, vaccination remains the most effective strategy for preventing and managing JEV infections (8), The neutralizing antibodies generated by vaccines play a crucial role in providing protection against JEV infection (46, 47), with the titer of these antibodies closely linked to the level of protection. To evaluate this, we measured the neutralizing antibody titers in mice vaccinated with the SA14-14-2 and SD12-F120 vaccines. Our results indicated that, following vaccination with SA14-14-2, the specific neutralizing antibody titer against the homologous genotype N28 (GIII) strain was higher than that against the sheep-derived SH2201 (GI) strain. Conversely, after vaccination with SD12-F120, the specific neutralizing antibody titer against the homologous SH2201 (GI) strain exceeded that against the heterologous SH2201 (GI) strain exceeded that against the heterologous N28 (GIII) strain. These findings are consistent with previous studies showing that neutralizing antibody titers specific to GI viruses were higher than those specific to GIII viruses in pigs and mice vaccinated with GI vaccines (39).

Currently, the SA14-14-2 vaccine is primarily used for the prevention and management of JEV infections in both humans and pigs (16). However, its protective efficacy in sheep remains uncertain. Therefore, we employed a mouse model to evaluate the protective effects of the SA14-14-2 vaccine against the genotype I JEV SH2201, isolated from sheep. Mice that received either high or moderate doses of the SA14-14-2 vaccine demonstrated complete protection against the homologous genotype N28 (GIII) strain, but did not achieve full protection against the SH2201 (GI) strain, exhibiting protection rates ranging from 60% to 80%. These results indicate that the GIII live attenuated vaccine provides partial protection against the SH2201 strain isolated from sheep. In contrast, mice immunized with high or moderate doses of the SD12-F120 vaccine exhibited complete protection against the SH2201 (GI) strain. This observation is consistent with prior research suggesting that high and moderate doses of the SA14-14-2 vaccine or GIII inactivated vaccines confer complete protection to mice against homologous GIII infections, but do not fully protect against heterologous GI infections, which exhibit protection rates between 60% and 80% (39). Additionally, mice vaccinated with the SD12-F120VC vaccine at doses of 2000, 200, or 20 PFU displayed nearly complete immunity against the homologous SD12 (GI) strain (30).

To further evaluate the immunoprotective effect of the attenuated live vaccines SA14-14-2 and SD12-F120, viremia and tissue viral load were detected in mice after challenge. The results showed that mice in the high-dose SA14-14-2 vaccine group did not exhibit viremia or tissue viral load after challenge with the SH2201 strain. In contrast, mice in the medium and low-dose SA14-14-2 vaccine groups showed varying levels of viremia and tissue viral load after challenge with the SH2201 strain. On the other hand, the SD12-F120 vaccine provides effective immunoprotection against the SH2201 strain at both high and medium doses, effectively inhibiting the appearance of viremia and tissue viral load. Additionally, the low-dose vaccine group only showed detectable virus in a few mice. Mice immunized with the SA14-14-2 vaccine were effectively protected from encephalitic lesions in the brain when challenged with the homologous genotype N28 (GIII) strain, indicating good protection against the homologous strain. However, encephalitic lesions were still observed in the brain tissue of mice challenged with the heterologous SH2201 (GI) strain, suggesting that the protective effect of the vaccine against the heterologous genotype SH2201(GI) strain was weakened. Mice immunized with the SD12-F120 vaccine showed no encephalitic lesions after challenge with either the homologous genotype SD12 (GI) or the SH2201 (GI) strain, indicating strong immune protection against both strains. Overall, these results indicate that the attenuated live vaccine SA14-14-2 provides poor immunoprotection against the heterologous SH2201 strain (a genotype I JEV isolated from sheep). This highlights the urgent need to develop new vaccines or vaccination strategies to effectively address infections caused by different genotypes and host species.

Neutralizing antibody titers (expressed as GMT) were assessed in 64 serum samples from clinically convalescent sheep with Japanese encephalitis revealing protection against the SD12(GI) strain (GMT ≥ 10) when the neutralizing antibody titer in the sera ranged from 1:10 to 1:20 against the SH2201 strain. When the neutralizing antibody titer against SH2201 was ≥ 1:40, it provided protection against both the SD12 (GI) and N28 (GIII) strains, with a 100% seropositivity rate for both strains. This finding parallels previous results indicating that neutralizing antibodies induced by a mouse brain-derived formalin-inactivated Nakayama vaccine can protect against prevalent GIII and GI viruses (26). Serum specimens capable of neutralizing the Nakayama virus were stratified by PRNT_50_ titers of 10, 20, 40, 80, 160, and ≥ 320, with specific GMTs calculated for the GIII CJN and GI TC2009-1 viral strains. This analysis showed that the GMT reached the presumed protective threshold (PRNT_50_ = 10) against CJN and TC2009-1 viruses when the neutralizing titer against the Nakayama virus was 1:20 or 1:80, respectively (26). Furthermore, pigs vaccinated with the SA14-14-2 vaccine had neutralizing titers of 1:20 or 1:40, reaching the protection threshold against N28 (GIII) and SD12 (GI) (PRNT_50_ = 10) (39). These results suggest that some degree of JEV infection does exist clinically in sheep. High titers of neutralizing antibodies not only enhance immunoprotection in sheep, but also provide comprehensive defense against various JEV strains, underscoring the vaccine’s effectiveness and the immune status of the sheep.

In conclusion, Japanese encephalitis virus (JEV) infection does occur in sheep in clinical settings. The SA14-14-2 vaccine provides partial protection against the SH2201 (GI) strain. To further evaluate the protective efficacy of existing vaccines in sheep, more comprehensive studies involving this host species are essential. Strengthening effective surveillance and control of measures for JEV in sheep is crucial to limit the virus’s spread within sheep populations and reduce the risk of human infection.

## Data Availability

The datasets presented in this study can be found in online repositories. The names of the repository/repositories and accession number(s) can be found in the article/**Supplementary Material**.
