# Supplementary material for: Partial protective efficacy of the current licensed Japanese encephalitis live vaccine against the emerging genotype I Japanese encephalitis virus isolated from sheep
Source: Front Immunol. 2025 Feb 13;16:1513261. doi: 10.3389/fimmu.2025.1513261 (PMC11865068; doi:10.3389/fimmu.2025.1513261)
Supplement: Supplementary file 1 [file DataSheet1.docx]

Supplementary Material

# Supplementary Table

**Supplementary Table 1. Information of JEV strains**

| Strains | | Genotype | 50% lethal dose (LD_50_)* | References |
| --- | --- | --- | --- | --- |
| SA14-14-2 | | III | NA | (1) |
| N28 | | III | 10^3.00^ | (1) |
| SD12-F120 | | I | NA | (2) |
| SD12 | | I | 10^2.70^ | (1) |
| SH2201 | | I | 10^1.35^ | This study |
|  |  |  |  |  |


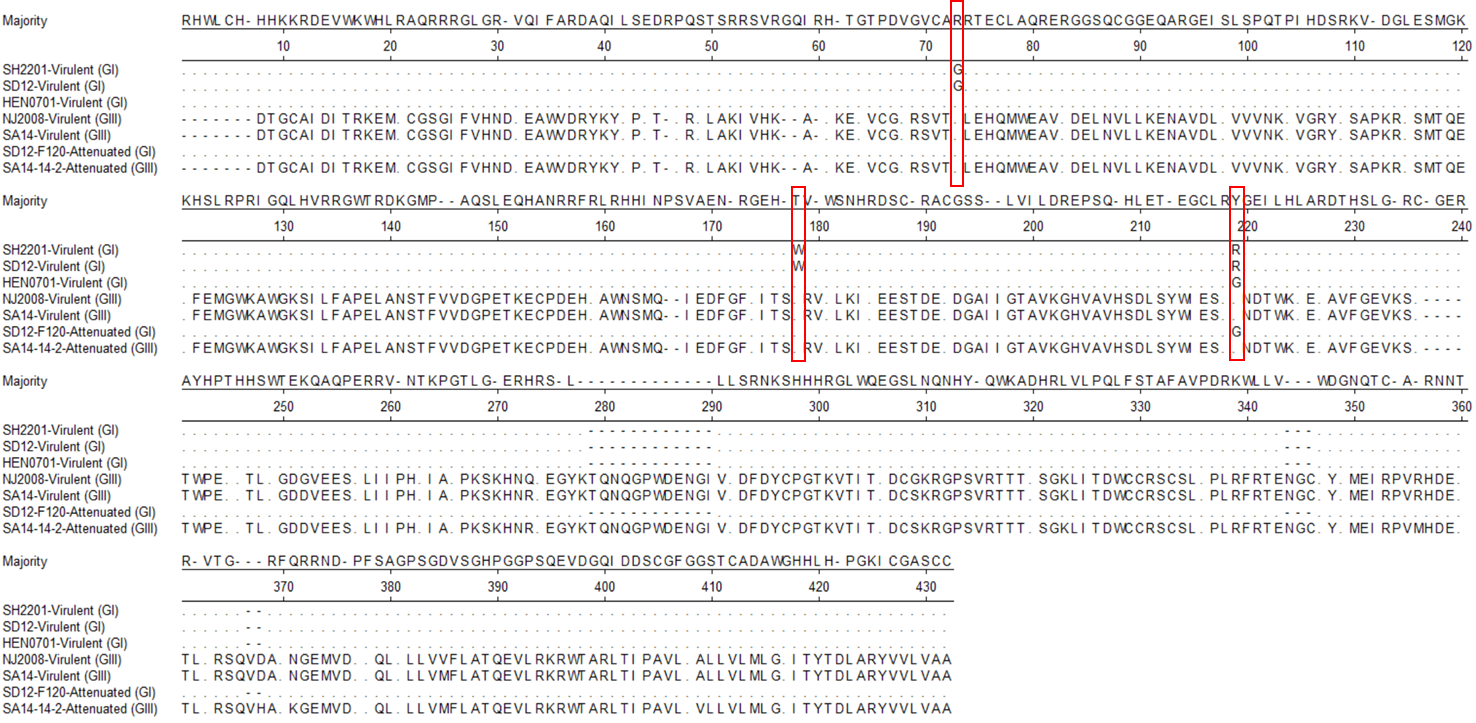


**Supplementary Figure 1. Comparison of amino acid sites of NS1 protein between JEV strains.** The amino acid variations between SH2201 and other JEV strains are boxed in red.

**Supplementary References**

1. Wei JC, Wang X, Zhang JJ, Guo S, Pang LL, Shi K, et al. Partial Cross-Protection between Japanese Encephalitis Virus Genotype I and Iii in Mice. *Plos Neglect Trop D* (2019) 13(8). doi: ARTN e000760110.1371/journal.pntd.0007601.

2. Anwar MN, Guo S, Xin W, Hameed M, Wahaab A, Ma XC, et al. Adaptation of a Live-Attenuated Genotype I Japanese Encephalitis Virus to Vero Cells Is Associated with Mutations in Structural Protein Genes. *Virus Res* (2021) 292. doi: ARTN 19825610.1016/j.viruses.2020.198256.
